# Changes in vessel density patterns assessed with OCTA in patients with diabetic macular edema treated with anti-VEGF therapy

**DOI:** 10.1007/s00592-024-02290-5

**Published:** 2024-05-27

**Authors:** Juan Santamaría, Estefanía Cobos, Marc Biarnes, Josep María Caminal, Ramon Rodriguez-Leor, Rahul Morwani, Manel García-Mendieta, Daniel Lorenzo, Pere García-Bru, Luis Arias

**Affiliations:** 1 Department of Ophthalmology, Hospital Universitari de Bellvitge, Carrer de La Feixa Llarga, S/N, 08907 L’Hospitalet de Llobregat, Catalunya Spain; 2 Institut de La Màcula, 08022 Barcelona, Spain; 3 Ophthalmology Department, Clínica Teknon, Barcelona, Catalunya Spain; 4 OMIQ Research, 08915 Sant Cugat del Valles, Spain; 5 Ophthalmology Oslo, Oslo Universitetssykehus Rikshospitalet, Oslo, Norway; 6 Hospital de Sant Pablo y Santa Tecla, 43003 Tarragona, Catalunya Spain; 7 Facultat de Medicina i Ciències de la Salut, Universitat de Barcelona (UB), c. Casanova, 143, 08036 Barcelona, Spain

**Keywords:** Diabetic macular edema, Vessel density, Optical coherence tomography angiography, Anti-VEGF inhibitors

## Abstract

**Aims:**

To determine the presence of sectoral changes in vessel density (VD) patterns induced by vascular endothelial growth factor inhibitors (anti-VEGF) in patients with diabetic macular edema (DME) using optical coherence tomography angiography (OCTA).

**Methods:**

Prospective, interventional study. A total of 43 patients (63 eyes) were initially enrolled in the study. We performed swept source (SS) OCT and sectorial OCTA measurement to determine parafoveal VD at baseline and after six months of anti-VEGF treatment. In the locations with statistically significant differences in VD between baseline and month 6, we performed univariate and multivariate analyses to determine which, if any, of the baseline variables were associated with the observed changes.

**Results:**

A total of 34 patients (48 eyes) were included in the final analysis. Mean VD decreased from baseline to month 6 (from 45.2 (± 3.5) to 44.6 (± 3.2) % in the SCP and from 50 (± 3.3) to 49 (± 3.9) % in the DCP). The only significant changes in VD were observed in the nasal sector of the deep capillary plexus, with a decrease of 2.9% (*p* = 0.001). On univariate and multivariate analyses, the only variable significantly associated with changes in VD in the nasal sector after 6 months of treatment was baseline VD in the same sector.

**Conclusions:**

Anti-VEGF therapy has a small impact on VD values over time. These variations observed after treatment seems to be related to changes over areas of vascular anomalies and displaced vessels adjacent to cystic areas, with no significant changes over ischemic areas. No correlation was observed between this trend and other clinical baseline features.

## Introduction

Diabetic retinopathy (DR) is characterized by microvascular changes (loss of capillary perfusion and damage to the blood-retina barrier), which can lead to the development of retinal ischemia and intraretinal exudation. These alterations are directly associated with disease severity and progression.

DR has several potential complications, including vitreous hemorrhage, diabetic macular edema (DME), and macular ischemia. [1, 2]

Currently, the gold standard technique to identify leaking vessels and ischemia (macular and peripheral) is fluorescein angiography (FA). However, this imaging technique has several important drawbacks: it is time-consuming, invasive, and may cause complications. [3]

By contrast, optical coherence tomography angiography (OCTA is a noninvasive technique that allows clinicians to visualize both the superficial and deep capillary plexus separately, which is not possible with FA.

In OCTA, vessel density (VD) is defined as the proportion of the vessel area showing blood flow relative to the total area measured. [4–6] Several studies have found that eyes with DR and DME have lower VD than controls, and that this characteristic is directly associated with the risk of retinopathy progression and the likelihood that treatment will be needed in the near term. [7, 8]

The effect of VEGF inhibition on macular vascularization during DME treatment remains controversial. While some studies have reported an improvement in macular non-perfusion areas following anti- VEGF treatment [9–11], other studies have not found any changes in macular VD even after three intravitreal injections. [12, 13] Moreover, two case reports reported significant visual deterioration after anti-VEGF treatment, attributing this phenomenon to VEGF pathway blockage during macular reperfusion. [14, 15]

At present, OCTA is not widely used to assess DME, mainly due to inaccurate segmentation, difficulty in differentiating cysts from areas of non-perfusion, and the lack of standardized vascular quantification methods. [16] In previous studies [12, 13, 17], VD was evaluated as a mean value for the superficial retinal capillary plexus (SCP) and deep retinal capillary plexus (DCP) respectively.

We hypothesize that the coexistence of ischemia and structural vascular anomalies such as telangiectatic vessels and microaneurysms, which are prone to leakage, may lead to different pathologic VD patterns in the same eye. If true, this would suggest that calculating a mean value for the whole macular area is the wrong approach, as it could overlook the presence of focal changes in some areas but not others. In this context, the aim of this prospective study was to evaluate both the mean and sectoral changes in macular VD after anti-VEGF treatment in eyes with DME and to assess the correlation between these changes and other clinical baseline features with a recognized prognostic value.

## Materials and methods

This prospective, interventional study was conducted at the Bellvitge University Hospital (Barcelona, Spain) from May 2018 to November 2020. All patients signed an informed consent form before enrollment. All patient-related data were anonymized for this analysis. The research protocol adhered to the principles of the Declaration of Helsinki and was approved by the Clinical Research Ethics Committee at Bellvitge University Hospital.

### Study population

We estimated the required sample size of the study. The assumptions included an α = 0.05, a β = 0.20 (for a power of 80%), a difference between pre/post periods of 2%, with a standard deviation (SD) of 5% and a discontinuation rate of 10%, with a two-tailed contrast. These assumptions led to a recruitment of at least 55 eyes for the study. The GRANMO software was used for these calculations. A total of 43 patients (63 eyes) were initially enrolled in the study. A comprehensive medical history and ophthalmological evaluation were performed in all cases.

The following data were collected at baseline: age; sex; type of diabetes; glycated hemoglobin (HbA1c) levels; lens status; and stage of retinopathy, which was recorded as either non-proliferative diabetic retinopathy (NPDR; mild, moderate, severe) or proliferative DR (PDR). Best-corrected visual acuity (BCVA) was determined according to the standard Early Treatment Diabetic Retinopathy Study (ETDRS).

Protocol Intraocular pressure (IOP) was also assessed. Color fundus photography, and OCT and OCTA imaging were performed after pharmacological mydriasis.

Inclusion criteria were as follows: age ≥ 18 years; treatment-naïve; and diagnosis of center-involving DME (defined as retinal thickening, intraretinal cysts, hyperreflective foci, or subretinal fluid documented by OCT, regardless of DR severity). Exclusion criteria were: BCVA < 35 ETDRS; vitreomacular traction or tractional retinal detachment on OCT; choroidal neovascularization or any other retinal vascular disease; poor quality OCTA images (signal strength intensity [SSI] < 40 due to media opacity or significant motion artifacts); previous history of any macular treatment (e.g., intravitreal anti-VEGF, corticosteroids, laser therapy, or vitrectomy).

All patients received three consecutive monthly injections of an anti-VEGF agent (ranibizumab or aflibercept) and continued with a bimonthly treatment regimen. BCVA and OCTA were performed at baseline and after 6 months of treatment. A normative database of macular VD values in healthy subjects for OCT Triton [18] was used to establish the mean normal value for each quadrant. Pre- and post- treatment VD were obtained to assess the deviation from normal values at baseline and post-treatment changes.

### Image acquisition

The swept-source (SS) OCT Triton (Topcon Corporation, Tokyo, Japan) was used to perform the SS-OCT and SS-OCTA measurements. This device uses a central wavelength of 1050 nm with an axial resolution of 8 μm, a transverse resolution of 20 μm, and a scanning speed of 100 000 A-scans per second. The OCTARA image processing algorithm was used. The SMARTTrack™ eye tracking technology was used to ensure that all follow-up OCTA exams were performed in the same position as in previous exams. A 6 × 6 mm scan in “angio-macula” mode was automatically centered at the fovea.

### OCT parameters

The following pre- and post-treatment parameters were recorded: central macular thickness (CMT) measured within 1 central mm; disorganization of inner retinal layers (DRIL); ellipsoid zone disruption, defined as discontinuity of the ellipsoid zone line within a 1.5-mm diameter of the fovea; hyperreflective retinal spots; and subfoveal neuroretinal detachment. These features were graded by two independent, experienced readers; in case of disagreement, the senior reader’s grading was used.

### OCTA parameters

Automated segmentation was used to evaluate the SCP and DCP. Segmentation errors were corrected manually. The SCP was segmented from 2.6 μm beneath the internal limiting membrane to 15.6 μm beneath the interface of the inner plexiform layer (IPL) and inner nuclear layer (INL). The DCP was segmented from 15.6 μm beneath the IPL/INL to 70.2 μm beneath the IPL/INL. VD measurements of the SCP and DCP were calculated automatically using the built-in software (OCTA-ratio analysis). VD measurements were made separately in four parafoveal subfields (Superior, inferior, nasal, and temporal) using an ETDRS grid overlay comprising the two inner rings.

### Statistical analysis

Descriptive statistics were used to characterize the sample. Depending on the distribution, means with standard deviation (SD) or medians with interquartile range (IQR) were used to describe quantitative variables. Categorical variables were described as number (percentage).

The relationship between baseline characteristics and baseline VD was evaluated using scatterplots with linear and LOWESS regression lines; the Pearson correlation coefficient (r) was determined for quantitative variables and either the independent t-test/Mann–Whitney or ANOVA/Kruskal–Wallis were used to compare quantitative variables between groups, depending on distribution normality.

We compared VD levels at baseline and at month 6 in the capillary plexus (SCP and DCP) and by location (superior, inferior, nasal and temporal sectors) using paired Student’s t or Mann–Whitney tests, as appropriate. In locations in which the difference between baseline and month 6 were statistically significant, we determined whether there was a significant association between the change in VD and the other variables analyzed. To do so, we applied both univariate and multivariate linear mixed effects models, which account for the correlation between eyes of a given individual in bilateral cases [19]. For this analysis, we used robust standard errors, an independent covariance structure and maximum likelihood as the estimation method. OCT categorical features were graded by two experienced ophthalmologists; the kappa coefficient was used to assess the degree of agreement between them.

The Stata IC software program, v. 15.1 (StataCorp; College Station, USA) was used to conduct the statistical analyses. A two-tailed *p*-value < 0.05 was considered statistically significant. We used the STARD checklist when writing our report. [20]

## Results

The final analysis included 48 eyes of 34, baseline features of the sample are shown in Table 1, 2**. Shows Pre- and post-treatment comparison of selected OCT features.**

**Table 1:** Baseline characteristics of study participants

Systemic features	Mean (SD) or n (%)*
Age, years	63.7 (± 10.5)
Sex, male	24 (70.4)
Type II diabetes	32 (94.1)
HbA_1C_	7.86 (± 2.04)
DR severity: Mild NPDR Moderate NPDR Severe NPDR PDR	4 (8.3) 20 (41.7) 6 (12.5) 18 (37.5)
Ocular features	
BCVA, letters	69.7 (11.2)
Macular thickness, µm	379.0 (98.6)
Treatment: Aflibercept Ranibizumab	33 (68.8) 15 (31.3)
Phakic	39 (81.3)
Cataract: None N0 N1 N2 N3 PSC	4 (10.3) 12 (30.8) 16 (41.0) 4 (10.3) 2 (5.1) 1 (2.6)

*BCVA* best-corrected visual acuity *DR* diabetic retinopathy *HbA1C* glycated hemoglobin *NPDR* non-proliferative diabetic retinopathy *PDR* proliferative diabetic retinopathy *PSC* posterior subcapsular cataract *SD* standard deviation

**Table 2:** Pre- and post-treatment comparison of selected OCT features. Measurements represent percentage unless for CMT, which is median (interquartile range). * Statistically significant differences. CMT: central macular thickness; DRIL: disorganization of retinal inner layers

OCT Feature	Pre-treatment	Post-treatment	*p*-value
CMT, microns	329 (97)	291 (85)	< 0.0001*
DRIL	14.6%	16.7%	0.78
Ellipsoid zone disruption	12.5%	6.3%	0.29
Hyperreflective foci	91.7%	85.4%	0.34
Subretinal fluid	20.8%	6.3%	0.04*
Intraretinal fluid	100%	89.6%	0.02*

Six months after completion of antiangiogenic therapy, the median (IQR) BCVA decreased slightly (*p* = 0.16) from 69.7 (15) to 68.5 (18) letters; median (IQR) macular thickness decreased significantly (*p* = 0.0001) from 354.5 (82) to 291 (85) μm. Table 3 shows the changes in VD between baseline and month 6 by location and plexus (SCP and DCP). VD generally decreased from baseline to month 6, but statistically significant differences were observed only for the nasal sector, with a 2.9% decrease (*p* = 0.001), and a small, borderline significant decrease in overall mean VD in the DCP of 1.1% (*p* = 0.055) (Table 3).

**Table 3:** Changes in vessel density between baseline and month 6 in the SCP and DCP by sector

	Superficial capillary plexus				Deep capillary plexus			
Sector	M0	M6	Diff	*P* value	M0	M6	Diff	*P* value
	%				%			
Superior	46.6 (3.8)	45.8 (4.4)	− 0.7	0.28	52.5 (5.9)	51.4 (5.6)	− 1.1	0.22
Inferior	46.0 (5.0)	45.7 (4.7)	− 0.2	0.77	50.4 (4.9)	50.0 (5.4)	− 0.4	0.71
Nasal	43.8 (4.3)	43.2 (3.9)	− 0.7	0.27	50.2 (4.8)	47.4 (5.3)	− 2.9	**0.001**
Temporal	44.6 (4.6)	43.7 (3.6)	− 0.9	0.16	47.0 (5.6)	47.0 (5.9)	0.0	0.99
Mean	45.2 (3.5)	44.6 (3.2)	− 0.7	0.16	50.0 (3.3)	49.0 (3.9)	− 1.1	0.055

*SCP* superficial capillary plexus *DCP* deep capillary plexus *Diff* difference (month 6 – month 0) *M* month

As Table 4 shows, the only significant associations between baseline characteristics and changes in VD at month 6 were observed in the nasal sector in the DCP. On the univariate analyses, baseline VD was the only variable significantly associated with changes in VD in the nasal sector at 6 months. On the multivariate analyses, this significant association was independent of age, sex, type of diabetes, HbA1C level, retinopathy severity, crystalline status, baseline BCVA, macular thickness, and intravitreal treatment. The effect size was slightly higher (absolute values) on the multivariate analyses (−0.79%, 95% CI −1.01 to −0.57; *p* < 0.001) compared to the univariate analysis (Table 4), suggesting that for each 1% increase in baseline VD in the nasal sector, there was a decrease of almost 0.80% in VD after 6 months.

**Table 4:** Univariate and multivariate linear mixed models evaluating the association between baseline features and changes in nasal VD. Statistically significant differences are shown in bold

	Univariate			Multivariate		
	β	95% CI	*p*-value	β	95% CI	*p*-value
Age, 1 year	− 0.07	− 0.26 to 0.12	0.46	0.00	− 0.26 to 0.25	0.97
Female	0.54	− 3.53 to 4.60	0.80	3.12	− 0.42 to 6.65	0.08
DM II	− 1.82	− 5.34 to 1.70	0.31	− 2.58	− 9.44 to 4.29	0.46
HbA_1C_, 1%	0.37	− 0.27 to 1.00	0.26	0.22	− 0.47 to 0.91	0.53
PDR	− 0.11	− 3.71 to 3.50	0.95	− 2.04	− 5.54 to 1.47	0.26
Phakic	0.56	− 4.74 to 5.86	0.84	− 0.87	− 5.80 to 4.05	0.73
BCVA, 1 let	− 0.02	− 0.18 to 0.14	0.80	− 0.06	− 0.17 to 0.05	0.32
CMT, 1 μm	0.00	− 0.02 to 0.02	0.97	− 0.01	− 0.03 to 0.01	0.45
BL − VD, 1%	− 0.73	− 0.96 to − 0.50	** < 0.001**	− 0.79	− 1.01 to − 0.57	** < 0.001**

*BCVA* best-corrected visual acuity *BL-VD* baseline vessel density *CI* confidence interval *DM II* diabetes type II *HbA*_*1C*_ glycated hemoglobin *Let* letters *CMT* central macular thickness *PDR* proliferative diabetic retinopathy

## Discussion

Ischemia and exudation due to vascular leakage often coexist in DME. [21] Recent studies support the idea that the former and the latter can be differentiated by their VD patterns. [22] Ischemic areas, which are due to capillary dropout are characterized by a reduction in mean VD values; by contrast, exudative areas have higher mean VD values due to vascular tortuosity, capillary telangiectasias, microaneurysms and displaced vessels secondary to cystic changes.

Our hypothesis is that calculating a mean value for the whole macular area, where this high and low VD coexist, may overlook the changes produced by treatment if any. Therefore, a sectoral approach may be more sensitive for detecting slight regional variations.

Regarding the effect of anti-VEGF drugs, several authors have reported changes in vascular blood flow (vasoconstriction and reduced flow) after intravitreal injections. Toto and colleagues [23] used laser speckle flowgraphy to assess blood flow, finding that intravitreal ranibizumab (IVR) decreased blood flow in the optic nerve head and in the peripapillary retinal vessels and that this decrease was associated with a reduction in CMT and improved BCVA. Fukami et al. [24] evaluated the effects of IVR in eyes with macular edema with branch retinal vein occlusion, finding that IVR induced transient vasoconstriction in the retinal arteries and veins and also reduced retinal blood flow. Okamoto et al. [25], found that choroidal vascular flow decreased significantly after a single IVR injection, and that this decrease was positively correlated with a reduction in CMT. Clearly, as those studies show, there is ample evidence that intravitreal treatment influences macular vascularization, mainly by reducing blood flow and leakage due to vasoconstriction.

In relation to the vascular impairment seen in DR, several studies have found that eyes with DR have larger arteriolar and venular caliber than controls, perhaps due to inflammation. [26, 27] In eyes with DME, the most significantly impaired retinal layer is the DCP, where abnormal capillary vortex patterns, cystoid spaces, and high flow microaneurysms (which are prone to leakage) are usually found adjacent to areas of retinal thickening. [24–27] These abnormalities are more likely to be found in the DCP than the SCP mainly due to the structure of this layer, which is predominantly composed of capillaries surrounded only by pericytes that are damaged by chronic hyperglycemia. [28]

After six months of treatment, we observed a trend towards lower VD values in all quadrants, although the only statistically significant decrease was in the nasal DCP. If we had used the Bonferroni method to adjust for multiple comparisons, the limit for statistical significance for changes in VD between baseline and month 6 would have been decreased to 0.05/10 = 0.005.

Importantly, changes in the nasal sector would still remain significant (Table 3; *p* = 0.001), suggesting that this finding is likely not due to chance alone since many comparisons were performed. These findings suggest that anti-VEGF therapy has only a minimal effect on vascular parameters assessed by OCTA, with a trend towards “healthy subjects’ values” especially in areas with increased VD.

**Fig. 1:**
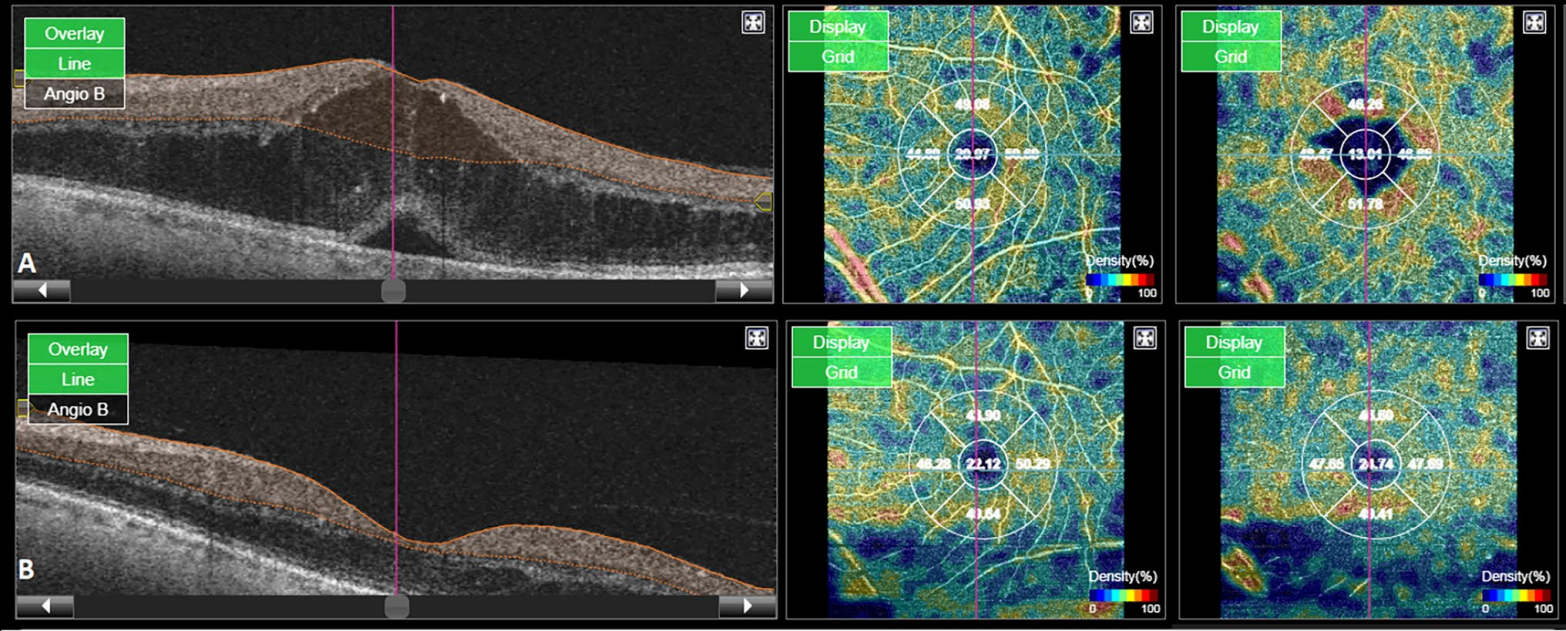
**A** Pre-treatment B-scan with the corresponding optic coherence tomography angiography (OCTA) vessel density (VD) map of the superficial (SCP) and deep capillary plexus (DCP). **B** Post-treatment B-scan with the corresponding optic OCTA VD map of the SCP and DCP. Note the reduction in the VD values mainly at the level of the DCP where the cystic changes were previously located, while little change is seen at the level of the SCP

These changes reached statistical significance in the DCP which is in concordance with the greater impairment seen in this plexus in DR as previously described. (Fig. 1).

Previous studies did not observe significant changes in VD after anti-VEGF or corticoid therapy [12, 17, 29, 30]. However, it is important to note that only a single mean VD value for the whole SCP and DCP was evaluated [12, 17, 29], and although Dastiridou et al. [30] also evaluated VD by quadrants, the total number of patients was less than half that of ours. By contrast, we evaluated the mean VD as well as sectoral changes, which provides a more comprehensive picture of these alterations. If VD is not measured separately, minor changes in areas with higher VD (due to microaneurysms and telangiectatic vessels) could easily be overlooked.

Carnota-Méndez P et al. [31] evaluated VD by quadrants, similarly to us, in 34 eyes treated with a single injection of dexamethasone, and found a significant reduction in VD at months 2 and 3 post-injection, mainly in the nasal quadrant, a finding that is in line with our results. Moreover, Toto L et al. [21] and Sugimoto M et al. [32] both found that vessels in the peripapillary area appear to be especially sensitive to anti-VEGF levels, causing a reduction in blood flow and vasoconstriction of arteries and veins, which would explain the changes in VD in the nasal DCP in our study and the study carried out by Carnota-Méndez P et al. In fact, those findings also support the notion that a reduction in vascular abnormalities (especially in the DCP) secondary to anti-VEGF treatment could normalize VD by reducing vessel diameter and the number of microaneurysms in areas with higher VD.

However, this variation in VD values could also be caused by changes in suspended scattering particles in motion (SSPiM) [33] SSPiM has been defined as an unusual extravascular signal on OCTA associated with the presence of hyperreflective material at the border of fluid spaces. This hyperreflective area may indicate the presence of subclinical extravasation of lipids and proteins secondary to blood-retinal barrier breakdown. (Maltsev DS et al. [34] evaluated the impact of SSPiM on VD in eyes with DME, finding that it was associated with artifactually increased VD in the foveal and perifoveal regions of the DCP. If anti-VEGF treatment reduces the SSPiM in cystic areas, that could result in an “artificial” reduction in VD values.

In our study, we did not find a significant increase in VD in areas with decreased values. This suggests that antiangiogenic treatment does not improve areas with capillary loss, which are due to permanent occlusion rather than leukostasis; therefore, the slight improvement observed in some cases might be due to a decrease in cystic spaces and repositioning of previously displaced vessels.

We found no direct association between changes in VD and the studied baseline features; however, higher baseline VD was associated with a greater decrease, perhaps because areas with higher baseline VD values may have a greater capacity to normalize, or simply due to regression to the mean. Therefore, it appears that changes in vascular parameters during anti- VEGF therapy are independent of previously known prognostic factors.

Regarding the slight decrease in BCVA, even though it did not reach statistically significance, it is not the usual response to therapy. We think it could be due to the wide range of baseline visual acuity. Most eyes either had good visual acuity with little room for improvement or had severe edema with low visual acuity and a higher probability of poor response to treatment.

### Strengths and limitations

This study has several limitations such as heterogeneity in the study eyes, wide range of visual acuity at baseline, limited sample size, duration and degree of loss of follow-up. However, the calculated sample size of 55 with up to a 10% of loss was met (48). By contrast, the study strengths include the prospective design, the homogenous treatment protocol, and the fact that all eyes were treatment-naïve. Another strength is the sector-by-sector approach to assessing VD. The advantage of this approach, which differs from most previous studies, is that it may be more sensitive in detecting small changes in macular vascularization.

## Conclusion

The findings of this study further support the idea that vaso-occlusive and exudative phenomena (characterized by abnormally low and high VD values, respectively) can coexist in the eyes of patients with DME. This variation in VD in different areas in the same eye imply that a single measure of VD for the whole eye may be misleading. For this reason, it probably makes more sense to assess macular vascularization sector by sector to obtain a more comprehensive picture.

Regarding the effect of anti-VEGF therapy on macular vascularization, even though ischemic and exudative patterns can be present in the same eye, the changes seen in VD after treatment could be explained by a positive effect over areas of vascular anomalies and displaced vessels due to cystic changes, which translates into a reduction of the abnormally high VD values towards the mean. In contrast, it does not appear to improve ischemia significantly, whose negative impact on visual prognosis is well-known. Ultimately, a better understanding of the effects of these drugs could provide reliable OCTA biomarkers to guide and improve clinical decision-making.

## Data Availability

All data generated or analyzed during this study are included in this article and its supplementary material files. Further enquiries can be directed to the corresponding author.
